# Innovative aspects of micropropagation in olive

**DOI:** 10.3389/fpls.2025.1561350

**Published:** 2025-04-15

**Authors:** Maurizio Micheli, Luca Regni, Anna De Carlo, Carla Benelli, Cristian Silvestri

**Affiliations:** ^1^ Department of Agricultural, Food and Environmental Science, University of Perugia, Perugia, Italy; ^2^ Institute of BioEconomy, National Research Council, Florence, Italy; ^3^ Department of Agriculture and Forest Science, University of Tuscia, Viterbo, Italy

**Keywords:** *Olea europaea* L., *in vitro* propagation, encapsulation, temporary immersion system, slow growth storage

## Abstract

The olive tree is one of the most iconic species within the Mediterranean Sea Basin. Countries bordering this sea enjoy a favourable climate that contributes to high-quality agricultural production for numerous horticultural species. The quality of the propagation material is one of the most important factors in determining the value of the final product, regardless of the cultivation-model, climate, and soil characteristics. Therefore, it is crucial to ensure genetic and sanitary certainty of vegetal/propagation material, which can be achieved through nursery productions. These goals are based on efficient propagation systems and methods to obtain high-biological quality plants. Over the last four decades, the application of biotechnology has introduced significant changes in the sector of nursery production. The Authors in this chapter aimed to present through their personal experimental experiences the latest advances in *in vitro* techniques and technologies that are revolutionizing the field of olive tree nurseries. While some of these methods are currently being employed, others are still undergoing research and development. Experts in this field firmly believe that all these techniques hold great practical value and have immense potential for high-quality nursery production.

## 1 Introduction

Over the last two decades, olive cultivation areas have expanded to regions across the globe (FAOSTAT, 2023) where the olive tree thrives and allows for the production of quality products (olives and oil). In addition, olive cultivation can leverage a diverse range of varieties and cultivation models, ranging from traditional grafting or cutting to innovative propagation (*in vitro* culture). Considering these assumptions, olive growers can choose from a wide range of cultivation techniques suited to their individual needs (Rosati et al., 2024) Biotechnological innovations have played a key role in modern nursery production by transferring research advancements into practical applications. In particular, *in vitro* culture techniques, such as micropropagation and *in vitro* conservation, have introduced a real industrial/commercial revolution. Many trees, shrubs, and even herbaceous species are very well adapted to these advanced propagation methods, which allow the quality of final productions to be elevated and introduce great practical advantages in the nursery field. These systems help overcome specific problems such as recalcitrance to rooting or graft disaffinity while keeping costs under control.

Despite the strong interest in the application of biotechnology to the olive tree, research in this species has hardly ever received adequate attention and resource support. Most studies have been conducted by researchers working limited in the countries of the Mediterranean basin, traditionally and historically the most interested in olive growing. This has reduced the expansion of research and technology transfer in this species, limiting the widespread adoption of *in vitro* propagation, despite increasing demand for high-quality plant material. However, biotechnological innovations hold great potential for olive nurseries, biodiversity preservation, and both short- and long-term conservation. *In vitro* techniques also facilitate the exchange of genetically and phytosanitarily certified plant material. This study aims to describe the experimental experiences conducted by the authors on *in vitro* culture techniques for olive trees. The main objective is to improve the efficiency of these techniques, whether they are currently being used in commercial production or to optimize them for future use. In this review, authors will discuss recent aspects of micropropagation, encapsulation technology for synthetic seeds production, slow growth storage, cultivation on liquid medium, and biotechnological tools mediated by tissue culture.

## 2 Micropropagation

*Olea europaea* L. is traditionally propagated vegetatively through various methods, including grafting scions onto seedlings, clonal rootstocks, or suckers, but the most prevalent method is the rooting of leafy stem cuttings under mist conditions (Bayraktar et al., 2020). However, the success of this technique is influenced by several factors such as season, cultivar, and the availability of healthy, viable material (Lambardi et al., 2013, 2023). For cultivars that are difficult to root, grafting remains the only effective method for clonal propagation. Nonetheless, grafting is more expensive, more complex, and requires specialized nurseries and skilled personnel (Fabbri et al., 2009; Lambardi et al., 2023).

To overcome the constraints related to the abovementioned traditional propagation techniques, *in vitro* propagation has been proposed and increasingly applied to olive as an alternative to agamic propagation technique (Bayraktar et al., 2020). Micropropagation, which involves growing olive cultivars from axillary buds, has proven successful and is used for commercial purposes in several Mediterranean countries, such as Italy and Spain (Fabbri et al., 2009; Lambardi et al., 2013; Sánchez-Romero, 2018). The main advantages of *in vitro* propagation consist of the high genetic and sanitary quality of the propagated material and the possibility to produce a large number of plants in a small space and in a short time, which facilitates the plant material exchanges between nurseries (Cardoso et al., 2018). However, the micropropagation of some economically important olive varieties remains difficult due to their recalcitrant nature, tissue oxidation, and challenges in obtaining sterile plant material and establishing *in vitro* shoot cultures (Lambardi et al., 2013). The success of olive micropropagation is highly dependent on the genotype, often resulting in low shoot proliferation rates, difficulty in rooting, high rates of post-transplanting losses, and, not least, the high cost of zeatin, the primary cytokinin used *in vitro* olive propagation (Grigoriadou et al., 2004; Sánchez-Romero, 2018; Regni et al., 2023).

In this context, one strand of research has been concerned with studying alternative approaches to optimize the protocol for olive tree micropropagation, focusing on the use of neem oil (Micheli et al., 2018; Regni et al., 2023), selenium (Se) (Regni et al., 2021), and zinc oxide nanoparticles (ZnO-NPs) (Regni et al., 2023). In particular, many efforts have been made to identify alternative cytokinin compounds or compounds with similar effects that can enhance the proliferation rate of olive tree explants and thereby lower the production cost per unit (Peixe et al., 2009; Micheli and Berenato, 2016). There is increasing interest in utilizing natural substances, sometimes referred to as “complex mixtures,” which, when added to *in vitro* cultivation medium, appear to improve proliferation rates. Among natural substances, neem oil, extracted from the seeds of *Azadirachta indica* tree, has been evaluated as a potential component of the medium in olive tree micropropagation. The beneficial effects of neem oil on *in vitro* olive plant regeneration were demonstrated for the first time by Micheli et al., 2018. The authors emphasized the benefits of neem oil to improve *in vitro* shoot proliferation in the olive Moraiolo cultivar. In this study, uninodal explants were cultured on Olive Medium (OM - Rugini, 1984) with different neem oil concentrations (0, 0.1, 0.5, and 1.0 mL L^−1^). The addition of 0.1 mL L^−1^ neem oil improved shoot regeneration, resulting in more vigorous and longer shoots, and a higher multiplication rate. Neem oil is a true nutritional supplement and, considering the large and varied number of molecules it contains, is considered a real “complex mixture” able to act as a plant growth promoter with effects similar to those of some growth regulators, such as gibberellins and cytokinins (Micheli et al., 2018). Another study by Regni et al. (2023) explored the addition of neem oil to the propagation medium, partially or completely replacing zeatin, during the proliferation and rooting phases of the Moraiolo cultivar. In the proliferation phase, media containing neem oil (0.1 mL L^−1^) and different zeatin concentrations (0, 1, 2, and 4 mg L^−1^) were tested. The results indicated that neem oil, combined with lower concentrations of zeatin (1 and 2 mg L^−1^), enhanced the number and length of adventitious shoots. This suggests that neem oil can significantly reduce the need for high concentrations of zeatin and lower production costs. For the rooting phase, agarised media and potting substrates were used, with shoots derived from standard and neem oil-enriched proliferation phase. The presence of neem oil in the rooting media did not directly enhance rooting, but the explants proliferated in a neem oil-enriched medium showed increased root number and length compared to controls (proliferated without neem oil). Further studies evaluated the effect of another natural substance in the *in vitro* propagation of ‘Moraiolo’: coconut water. It had previously been used by other authors in olive trees (Peixe et al., 2007), but never with this cultivar. The addition of 10% coconut water combined with three concentrations of zeatin (0, 2 and 4 mg L^-1^) to the OM nutrient substrate was studied in order to evaluate its effect on *in vitro* multiplication of uninodal explants of ‘Moraiolo’. The use of coconut water added to the medium OM without zeatin provided interesting results, stimulating the development of the highest number of new-formed shoots (14.2), which, however, were the shortest in length (less than 0.5 mm) in comparison to those obtained with the other treatments. On the other hand, the combination with 2 mg L^-1^ zeatin resulted in the same vegetative performances as those recorded with 4 mg L^-1^, i.e. the concentration usually used for ‘Moraiolo’ proliferation. This would already be enough to halve the cost of producing *vitro*-derived shoots in this olive cultivar (Micheli and Berenato, 2016).

Selenium (Se) known for its antioxidant properties and ability to improve plant growth and stress resistance has also shown promising in enhancing olive plants micropropagation (Regni et al., 2021). The effects of different Se concentrations (0, 10, 20, 40, and 80 mg L^−1^) were studied in four olive cultivars: ‘San Felice’, ‘Canino’, ‘Frantoio’, and ‘Moraiolo’. Results demonstrated that Se concentrations between 10 and 40 mg L^−1^ increased shoot lengths, fresh and dry weights of the proliferated explants in all cultivars. Se treatment also resulted in higher Se content in the explants, indicating efficient absorption and accumulation. However, the beneficial effects tended to diminish with successive subcultures, suggesting an adaptation effect.

The application of ZnO-NPs in olive tree micropropagation was also investigated to enhance growth and biochemical parameters (Regni et al., 2022). Biogenic ZnO-NPs, synthesized using *Lemna minor* L. extract, were added to the growth medium at concentrations of 0, 2, 6, and 18 mg L^−1^. Explants treated with 6 and 18 mg L^−1^ ZnO-NPs showed significant improvements in shoot number, fresh and dry weight, and chlorophyll content. Moreover, these ZnO-NPs concentrations increased carotenoid, anthocyanin, and total phenol content, as well as antioxidant activity. These findings suggest that ZnO-NPs, by enhancing the content of molecules involved in photosynthesis and plant growth, can significantly promote olive tree micropropagation.

In the context of innovative aspects of micropropagation in olive, the evolution of *in vitro* rooting techniques represents a significant advancement. Traditionally, *in vitro* rooting methods are now considered outdated, with the current trend favouring a more integrated approach that includes *ex vitro* rooting and acclimatization concurrently. This approach involves transferring mini-cuttings directly into pre-formed containers filled with commercial substrates composed of soil, peat, and perlite mixes. These containers are maintained in a controlled environment to facilitate a gradual transition from the highly controlled conditions of *in vitro* culture to the natural conditions found *in vivo*.

**Figure 1:**
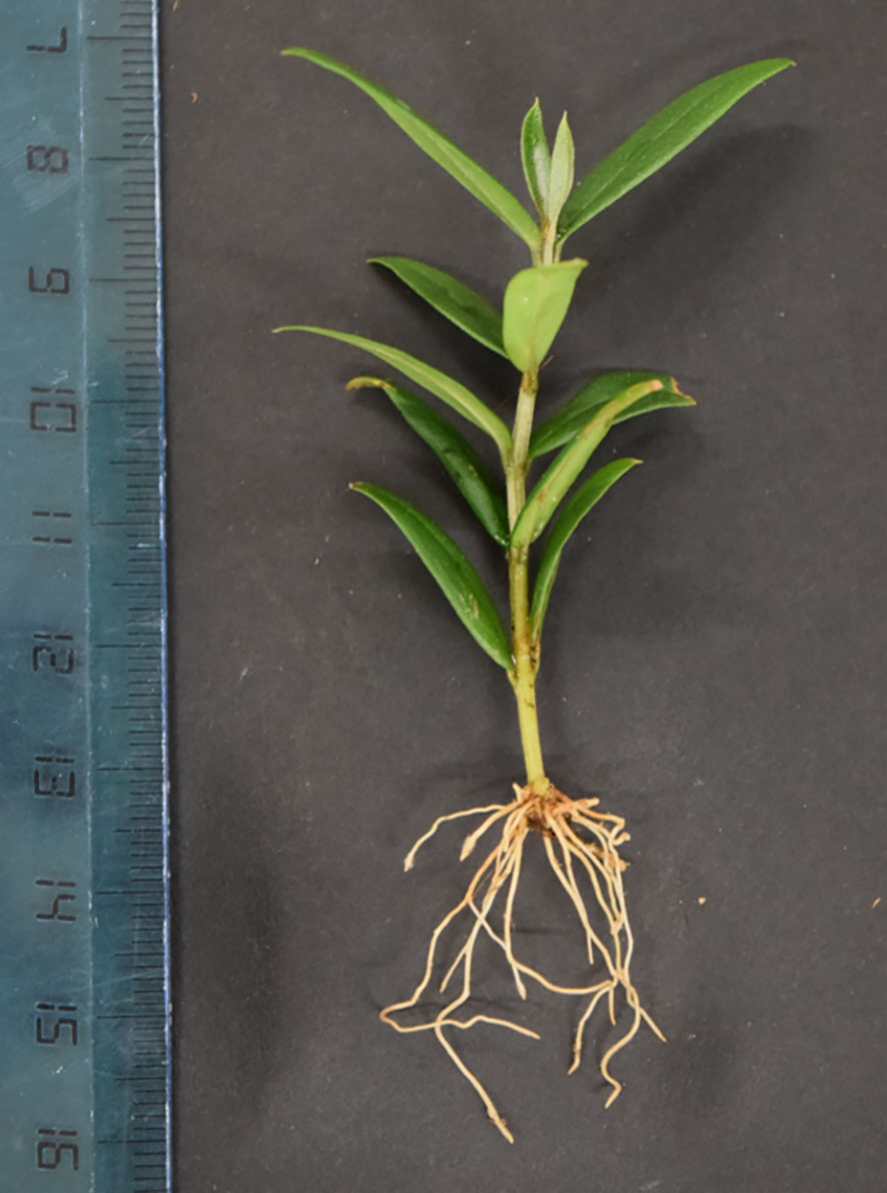
*Ex vitro* rooting of an olive mini-cutting.

For certain olive cultivars, such as ‘Sirole’ and ‘Frantoio’, this method has demonstrated exceptionally high rooting rates, approaching 100%. However, for cultivars that usually show resistance to traditional rooting methods, additional strategies are employed. One effective technique involves treating mini-cuttings with an auxin solution, such as a liquid indole-3-butyric acid (IBA) solution applied directly into the propagation containers. This pre-treatment, lasting 3 to 10 days prior to transplanting for rooting, has been shown to significantly enhance rooting success, achieving rates above 80% even in cultivars considered recalcitrant. The integration of rooting and acclimatization stages into a single step is a substantial advantage in modern olive micropropagation. This approach not only streamlines production processes but also enhances the quality of the resulting plant material. Mini-shoots rooted in this manner tend to develop robust, well-established root systems (**Figure 1**) that outperform those obtained through traditional *in vitro* rooting methods.

This shift towards integrated *ex vitro* rooting methods underscores ongoing advancements in olive micropropagation techniques, promising improved efficiency and higher-quality plant production.

## 3 Encapsulation technology

**Figure 2:**
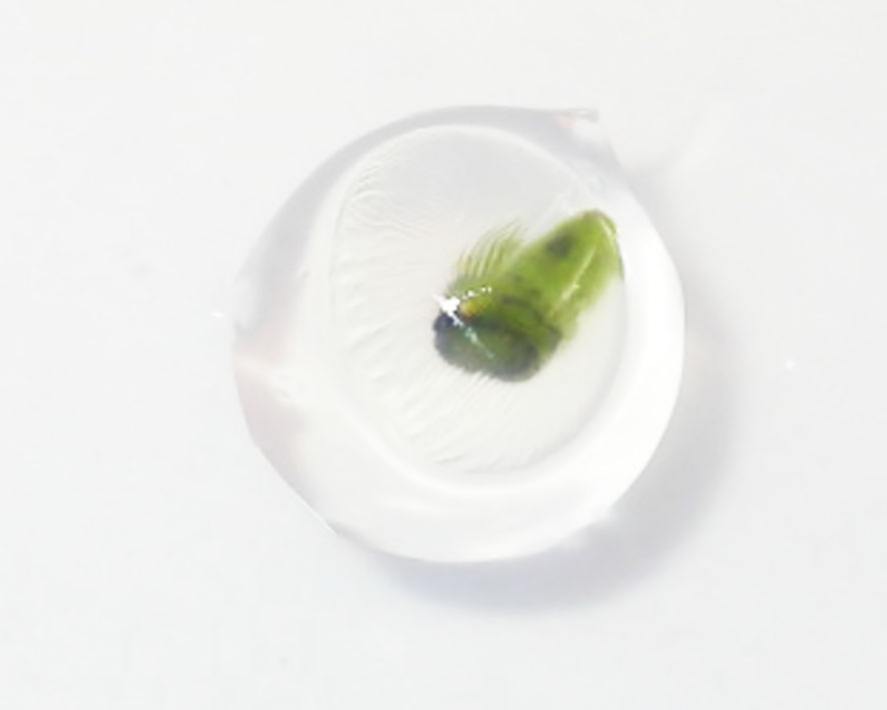
Development of olive shoot tip from calcium alginate bead.

As reported above, modern nurseries are increasingly adopting *in vitro* propagation techniques, such as micropropagation, which enable the mass production of high-quality plantlets with excellent genetic health, even from limited starting materials. This approach effectively addresses the rising costs associated with traditional methods by utilising innovative techniques and technologies that facilitate the rapid production and distribution of genetically uniform materials with significant agronomic or environmental value, such as commercial varieties, rootstocks of particular interest, restored plants, and genotypes that must be preserved *ex situ*. To simplify the management of micropropagated materials, especially during and after the acclimatisation phase, and limit the final costs, the encapsulation technology could represent a new productive tool. It combines the advantages of clonal propagation with the benefits of seed propagation, such as smaller size, ease of transport, and storage for short/medium/long-time (Micheli et al., 2022). This can help to meet the high expectations of nursery operators for innovation, which is a crucial factor in advancing the plant breeding sector (Standardi and Micheli, 2013). The concept of encapsulation was introduced by Murashige in 1978, who suggested covering the *vitro*-derived propagules with a calcium alginate-based gelled matrix, with a nutritive and protective function by using somatic embryos. These propagules can develop into complete plantlets when subjected to suitable environmental and nutritional conditions. However, the effectiveness of this technological innovation is closely linked to the availability of efficient and reliable protocols of somatic embryogenesis, in the absence of which the encapsulation has not yet been implemented for most *in vitro* cultivated species (Gantait and Kundu, 2017). To overcome this limit, research by Bapat and Redembaugh (1993) and Leathers et al., 1995 expanded the variety of propagules including non-embryogenic types. This led to the development of a new classification of encapsulating propagules, including microbulbs, microtubers, rhizomes, protocorms, meristemoids, tissue or organ fragments, meristematic apices, root sections, and both apical and axillary buds. They can be easily obtained through shoot proliferation or direct organogenesis (Lambardi and Micheli, 2019). Depending on the species, the propagules capable of developing both shoots and roots simultaneously are referred as bipolar propagules useful to produce *synthetic seeds*. Others, known as unipolar propagules, can be encapsulated obtaining *beads* able to develop either shoots or, less commonly, roots, as they possess only the apical or root meristem (Benelli et al., 2017). A common example of unipolar propagules is represented by microcuttings, which are uninodal sections of shoots derived from tissue culture, typically measuring 3 to 5 mm in length, unable to develop roots spontaneously because possessing only axillary or apical buds. The possibility of obtaining synthetic seeds by unipolar propagules is strictly connected to stimulating an organogenetic process to develop the root system (Standardi and Micheli, 2013). The potential for using various propagules allows the application of encapsulation products for different goals. These include long-term conservation of germplasm through low-temperature storage or cryopreservation (Lambardi and Shaarawi, 2017; Lambardi and Micheli, 2019; Wang et al., 2020) and the exchange of valuable rare for hybrids and elite genotypes (Benelli et al., 2017). Additionally, it simplifies the management of mass clonal propagation products in different plant species (Gantait and Kundu, 2017), particularly with the integration of automation systems for certain processing steps (Singh, 2018). Despite its promising potential, encapsulation is still not a widely used technology, particularly in relation to certain plant species. One such example is the olive tree, which has only recently been propagated *in vitro* for commercial purposes. The reduced number of effective protocols for regeneration through somatic embryogenesis, restricted to only a few more responsive olive varieties (Abd El-Zaher et al., 2024; Palomo-Ríos et al., 2021; Sánchez-Romero, 2021), limits the production of propagules suitable for creating synthetic seeds directly. In contrast, some researchers initially concentrated the studies on identifying the most suitable propagules for encapsulation technology in olive trees: they found that, in the absence of viable alternatives, microcuttings have been determined to be the most accessible explants (Standardi and Micheli, 2013). This type of propagules is usually excised from the elongated shoot axis at the end of an *in vitro* proliferation subculture. Each microcutting corresponds to a node bearing axillary buds or an apical one without leaves. When the nodes are directly subjected to encapsulation in calcium alginate solved in a nutrient matrix, beads are obtained: they are structures capable only of developing new shoots from the buds (**Figure 2**), without the regeneration of a root system (Standardi and Micheli, 2013). Some problems may occur and limit the sprouting ability of the beads, such as the axillary bud’s dormancy of some olive varieties (Micheli et al., 2018). To overcome this problem, single or combined treatments with gibberellic acid solutions (1 mg L^−1^) and short/medium-term storage (from 45 to 180 days) at low temperatures (4°C) have been proposed on ‘Moraiolo’ microcuttings (Micheli and Standardi, 2005; Micheli et al., 2007; Ikhlaq et al., 2010; Micheli et al., 2019), in addition to optimisation of the nutrient composition of the encapsulating matrix (artificial endosperm), in particular trying to identify the best concentration of sucrose (from 15 to 30 g L^−1^), an essential element for keeping the propagule alive and stimulating it to develop the new shoots (Micheli et al., 2019).

After improving the vegetative behaviour of the encapsulated microcuttings of ‘Moraiolo’, the next step was to induce their rhizogenesis capacity, thereby producing synthetic seeds using the unipolar nodes. In olive trees, as with other *in vitro* propagated species, adventitious root development usually relies on exogenous auxin treatments, specifically IBA (indole-3-butyric acid indole-3-butyric acid) and NAA (naphthaleneacetic acid) (Lambardi et al., 2023). The success of the treatment depends on genotype, kind of auxin, its concentration and the duration of the treatment (Allatif and Hmmam, 2022). Based on what has been studied for micropropagation, auxin treatment (dipping the microcuttings of olive in a 5 mg L^−1^ IBA solution enriched with 15 g L^−1^ sucrose and maintaining them for 24 h in darkness on rotator shaker at 100 rpm) was also found to be effective in inducing formation of root primordia in olive microcuttings before encapsulation (Standardi and Micheli, 2013; Micheli et al., 2019) or after by dipping the beads directly in the same auxin inductive solution (Micheli et al., 2006) to induce them to convert (Micheli et al., 2019). Further studies showed an interesting positive effect of cold treatment (4°C) alone or in combination with the IBA treatment on the conversion of the synthetic seed of olive (Micheli et al., 2019).

## 4 Temporary immersion systems

The use of liquid medium for *in vitro* culture has been investigated in detail over the years (Takayama and Misawa, 1981; Harris and Mason, 1983; Berthouly and Etienne, 2005). *In vitro* production of plant species using a liquid medium in bioreactors is a complementary strategy in order to overcome the limitations present in the *in vitro* system on conventional semi-solid media. In comparison with culturing on semi-solid media, larger containers can be used, and subculture times can be reduced to avoid intensive manual handling (De Carlo et al., 2021).

The liquid medium has also frequently been considered an ideal tool for biomass production as it reduces manual labor, facilitates the change of composition medium, beyond offering benefits in increased nutrient uptake, greater availability of dissolved oxygen, easier dispensing of the medium, automated scale up and process control, and more productivity (Leathers et al., 1995; Mirzabe et al., 2022). Bioreactors for liquid culture used in the past were not suitable for the propagation of plants, they were mainly developed for bacterial culture and did not consider the specific requirements of plants. After several tests, the Temporary Immersion System (TIS) was applied to plants and deemed a good practice (De Carlo et al., 2021). The novelty of TIS is due to its ability to allow a contact, partially or totally, programmable between the explants and the liquid medium. TIS bioreactors are periodic semi-automated or fully automated cultivation systems, based on alternating cycles of temporary immersion of the cultured plant tissue into the liquid medium followed by a dry period. Usually, the immersion period is shorter (a few minutes), whereas the dry period is prolonged (several hours); the whole system is controlled by pumps and timers to plan the different cycles.

The optimization of the suitable immersion time is the most critical parameter for system efficiency, and it must be monitored to avoid the hyperhydricity of culture; moreover, the choice of volume of nutrient medium and the size of the container also substantially improve efficiency (Etienne and Berthouly, 2002; Quiala et al., 2012; Carvalho et al., 2019; Garcìa-Ramìrez, 2023). Usually, TIS bioreactors are made with transparent glass or plastic vessels, so that the *in vitro* plants can be illuminated with external light (De Carlo et al., 2021).

Different type of TIS bioreactors are now available (Georgiev et al., 2014; De Carlo et al., 2021), consisting of one or two containers, and some of them are also equipped with additional option that permits the periodic replacement of the atmosphere within the culture container, which limits gas accumulation (mainly, CO_2_ and ethylene).

The positive effects of temporary immersion on micropropagation are reported on shoot proliferation and microcuttings, microtuberization and somatic embryogenesis in different species such as *Stevia rebaudia* (Takayama and Akita, 1994), *Solanum tuberosum, Coffea arabica* (Etienne and Berthouly, 2002), *Quercus suber* (Pérez et al., 2013) and *Crocus sativus* (Tarraf et al., 2024).

Although the TIS is a recent approach to micropropagation, several reports on woody plants are already available in the literature (Wilken et al., 2014; Akdemir et al., 2014; Carvalho et al., 2019), and it can be taken into consideration in developing new technologies for olive micropropagation.

In olive, the first investigation on TIS was carried out with a Greek olive ‘Chondrolia Chalkidikis’ (Grigoriadou et al., 2005). In this study, a custom made TIS bioreactor, called ‘NovelTIS’ was used, with uni-nodal olive shoots, as explants, and WPM medium with zeatin (20 µM), GA_3_ (10 µM), NAA (0.3 µM) and sucrose (2%). In this report, only one immersion period of 15 min every 8 hours was tested. This device showed good results, but without significant differences with respect to semi-solid medium, in terms of improvement of the new shoots number or average shoot length, at the end of subculture (30 days).

RITA^®^ system (VITROPIC, France), a semi-automatic TIS available on the market, was applied on ‘Canino’ olive shoots (Lambardi et al., 2006). This type of bioreactor combines a periodic immersion in liquid medium of shoot cultures with periodic air replacement by pneumatic transfer avoiding the accumulation of gases in the culture container. The explants used in this study were uni-nodal, bi-nodal and tri-nodal olive microcuttings with OM liquid medium added with mannitol (36 g L^−1^), zeatin (10 µM). Three immersion periods were tested (4min/2h; 16min/8h; 16min/16h). Overall, bi-nodal microcuttings of ‘Canino’ positioned horizontally in the RITA^®^ container and immersed in the liquid medium 16 min every 8 hours, performed better results, also when they were compared with bi-nodal microcuttings grown in semi-solid OM medium with the same composition reported above. In the same study, the best growth conditions recorded on cv. Canino were tested in RITA^®^ bioreactors on four olive cultivars Arbequina, Gentile di Larino, Frantoio and Ascolana Tenera. Promising improvement in average node number was observed in TIS for microcutting of ‘Frantoio’ and ‘Ascolana Tenera’, while the proliferation in ‘Gentile di Larino’ was similar to the control shoots growing in a semi-solid medium. Cultivar Arbequina showed a slight decrease compared to the control, but the shoots had an increase in length of internodes.

**Figure 3:**
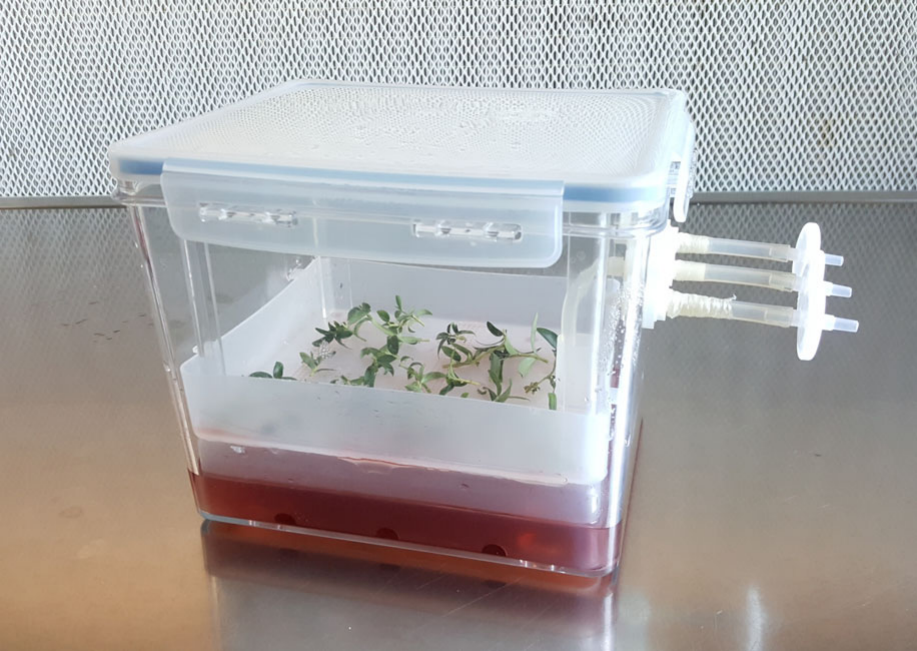
Temporary Immersion System by Plantform^TM^ bioreactor for olive shoots proliferation.

Peixe et al. (2007), in a study that aimed to simplify the olive micropropagation and reduce its cost, reported a short information about application of RITA^®^ on cv. Galega Vulgar without given specific detail on the used protocol; in results they asserted that this system leads to a reduction of hand-labour necessary for the systematic subcultures. Another type of TIS bioreactor applied on olive cv. Canino has been Plantform™ (Benelli and De Carlo, 2018), consisting of a single transparent container with a wide size that allows a greater number of plants in each unit and a controlled ventilation (**Figure 3**). In this research, the TIS showed good adaptability and better growth rates for olive shoots in comparison to conventional systems in glass jars with semi-solid medium. Significant differences in proliferation, shoot length and better vigour of shoots were noted when an immersion frequency of 8 min every 16 h and a ventilation of 15 min every 4 h were applied. In addition, the Plantform™ bioreactor allowed the use of a lower concentration of zeatin (5 µM, instead of 10 µM) without any change in the growth performance of the shoots. Zeatin is a fundamental hormone to ensure a good proliferation rate *in vitro* culture of olive (Lambardi et al., 2023) and its high cost has always penalized the micropropagation of this important species. The study emphasizes the possibility to achieve an efficient *in vitro* olive proliferation with lower production costs, maintaining high-quality olive shoots.

**Figure 4:**
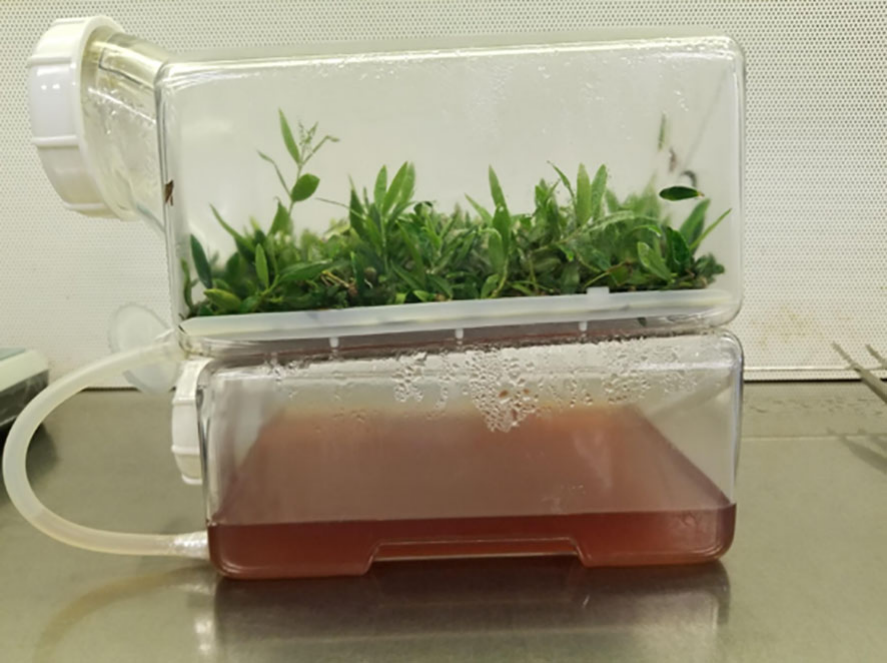
Efficient olive shoot proliferation in SETIS ^TM^ bioreactor.

Overall, the findings show that the TIS can represent a valid alternative to conventional systems *in vitro* olive propagation, resulting in a reduction of costs, labour and time for biomass production. In fact, several are the advantages of the culture in TIS. The size of recent bioreactors allows a larger number of plants in each unit which reduces labor for transferring plants; the enrichment of oxygen can be regulated, and the accumulation of detrimental gases can be avoided. Moreover, the acclimatization phase that generally causes great losses of plants can be facilitated by improving the stomata function in TIS, so the plants are easier to establish *ex vitro* (Martínez-Estrada et al., 2019; Bello-Bello et al., 2019; Mancilla-Álvarez et al., 2024). Comparative studies between the semisolid medium and the bioreactor culture, in general, revealed that shoot proliferation and growth were more effective in TIS system (De Carlo et al., 2021), as occurred in olive cultivar ‘Maurino’ when the shoots were cultured in SETIS bioreactor (**Figure 4**), this latter showed the best proliferation rate respect to conventional system.

The main drawbacks reported in TIS culture were the shoots hyperhydricity for some species, resolvable by applying the appropriate immersion cycles, and more difficult control of the microbial contamination due to the greater number of plants kept in the container (Dewir et al., 2014; Lotfi et al., 2020). The introduction of a new type of TIS bioreactor, called ElecTIS (http://www.explanta.com/bioreactor-electis/) is in progress in olive cv. Canino at the Laboratory *In Vitro* Cultures of IBE/CNR. This bioreactor is innovative compared to other TIS because it is not the liquid to move up and down, but an internal basket, containing the shoots, moves down into contact with medium by programmable pump. In this system a lower amount of air blown for the basket movement is necessary with minor risk of culture contamination. ElecTis has already been successfully applied on *Malus* (Sota et al., 2021) and *Rubus fruticosus* (Elazab et al., 2023) and offers good perspectives also for *in vitro* propagation of olive.

## 5 Slow growth storage

Many National and International programs are currently underway for the conservation of the large genetic variability of the olive tree in order not to lose interesting genotypes, while the current tendency is the cultivation of cultivars with more productive and adaptable to most environmental conditions in the new olive plantations (Rugini and Gutiérrez-Pesce, 2006).

At present, field clonal collection is the most widespread option for preservation of olive germplasm; however, this traditional method is expensive and subject to losses due to diseases, parasites, extreme environmental conditions, and economic efforts (Fabbri et al., 2009). To preserve diversity at all levels (genes, species and ecosystems), which are constantly evolving, and to make them always available, it is necessary to use or combine alternative conservation tools. For this reason, there is an increasing interest in the conservation of olive germplasm through methods involving *in vitro* culture technology.

Generally, *in vitro* approaches are considered promising for the conservation of plant biodiversity that includes the preservation of genetic resources of vegetatively propagated species, threatened plant species, taxa with recalcitrant seed, elite genotypes, and genetically modified/engineered material (Chauhan et al., 2019). Considering the olive tree, where the conservation of seeds is restricted to rootstocks, and the various cultivars are propagated vegetatively, the *in vitro* conservation, by means of Slow Growth Storage (SGS) for medium-term preservation and by cryopreservation for long-term preservation, can be a successful strategy complemented and integrated with traditional conservation methods. The SGS (also called ‘minimal growth’) is achieved by keeping explants in modified culture conditions that slow down cell metabolism and growth. At the end of the storage period, the cultures are transferred to optimal standard conditions to stimulate their proliferative recovery. This technique reduces markedly the frequency of periodic subculturing, compared to the standard intervals of 3-5 weeks (Reed, 1999).

Depending on the species, conservation in SGS can be extended from a few months to a year or more, without compromising the viability and the growth potential of the shoots and with less interference with the culture system therefore reducing the risk of contamination.

Research on many woody plants has shown that 12 months between subculture intervals seem to be a more realistic goal, but for some species, the period can be more extended (Benelli et al., 2022).

The growth reduction is generally obtained by modifying the culture medium and/or the environmental conditions. The growth and quality of the shoots maintained in SGS are affected by several factors, such as temperature, presence or absence of light, light intensity, composition of the growth medium (macroelements, carbohydrates, growth regulators, osmotically active compounds, growth retardants and antioxidants), the type of container and the type and physiological state of explant. The application of one or more factors contributes to obtain the maximum time of storage explants (Benelli et al., 2022).

The most used method to ensure slow growth storage, without compromising the quality of the shoots, is the maintenance of the explants at a temperature below that required for optimal growth of assessed species.

Medium-term preservation strategy is actually used for a large number of plant species, including several threatened species of tropical and temperate, in biodiversity conservation programmes and *in vitro*-banking (Ruta et al., 2020).

Commercial micropropagation laboratories also use this technique to maintain the mother plants and to expand the supply of species and cultivars and to achieve a better organization of their production. In fact, the technique allows a significant extension of the interval between subcultures, thus reducing the management (operative) costs of the culture of breeding and the risks of contamination during subculture.

Although SGS is often applied in commercial and *in vitro* culture laboratories for the conservation of fruit species, in the case of the olive its application is still very sporadic.

Gardi et al. (2001) observed a different behaviour in the shoots of several olive cultivars during SGS. ‘Frantoio’ was stored for 5 months at 6°C in the dark, showing 100% of survival and regrowth after conservation, while for shoots of ‘Ascolana tenera’ and ‘Moraiolo’, preserved in the same condition, the maximum regrowth potential was only up to 2 months.

Two olive cultivars (Leccino and Frantoio) were preserved for 8 months at 4°C on hormone-free OM medium (Olive medium; Rugini, 1984) under dark conditions (Lambardi et al., 2002). High rate of regrowth (up to 80%) was achieved in post conservation in standard culture conditions (recovery). On the contrary, the preservation of the shoots at 4°C under 8h photoperiod of light, has involved a drastic significant reduction in the percentage of shoot recovering for both cultivars: 15% (Leccino) and 10% (Frantoio). Extending the conservation to 12 months, although the survival rate remained satisfactory for ‘Leccino’ (66%), the percentage of regrowth was almost nil after 6 weeks of recovery. Moreover, the same study has tested innovative containers, called ‘Vitro Vent’ (Duchefa Biochemie BV, Netherlands), that allowed continuous ventilation and prevented the accumulation of volatile compounds inside the container. This non-forced ventilation reduces internal condensation and makes the containers very suitable for *in vitro* storage. Although the plants are maintained at conditions of reduced metabolism, gas composition inside the vessels, during SGS, can affect the storage period and shoot regrowth in post-conservation. In this context, Benelli et al. (2002) investigated the dynamics of carbon dioxide and ethylene accumulation in ‘Frantoio’ culture vessels during 8 months of olive SGS. Olive shoots were stored at 4°C in darkness into glass serum bottles (100 ml volume) containing hormone-free OM medium and sealed with air-tight vial rubber septa. During the storage period a rapid (a few weeks after the beginning of the SGS), and conspicuous (up to 70.000 μl/L after 8 months) accumulation of CO_2_ within the vessel was observed, while ethylene production was negligible. Therefore, the authors hypothesized a relationship between high levels of CO_2_ and poor recovery of shoots after SGS.

The possibility of storing olive explants in capsules (or synthetic seeds) of Ca-alginate matrix containing a nutritive medium has been tested for short period as aseptically exchange of plant material between laboratories over long distances without danger of any loss and reducing the phytosanitary and quarantine problems. Micheli et al. (2007) investigated the storage of encapsulated olive microcuttings (one node with two axillary buds) of ‘Moraiolo’. The capsules were stored with a simple procedure in plastic cuvettes with an artificial endosperm solution to maintain the relative humidity during the SGS period. After SGS, the capsules were transferred into glass vessels with OM medium to evaluate the recovery. The olive capsules maintained a high ability to regrowth after storage of 15 and 30 days at 18°C and 4°C. In particular, after 30 days at 4°C the average number of shoots sprouted per capsule was similar to the control, as well as the multiplication rate.

Encapsulated microcuttings of ‘Canino’, ‘Moraiolo’, ‘Ascolana tenera’ and ‘Dolce Agogia’ on semi-solid medium were stored at 4°C successfully for 30 days (Gardi et al., 1999). The explants showed a viability rate between 47 and 100% immediately after storage, depending on the cultivar, but poor results in terms of regrowth in post conservation were observed.

The conservation of synthetic seeds of ‘Moraiolo’ cultivar was also tested at 4°C and 21°C for different periods (15, 30, 45 and 60 days) (Ikhlaq et al., 2010). The best condition in terms of parameters evaluated (germination percentage, number of shoots per explant, shoots length, number of nodes, rooting percentage, number of roots per explant and root length) was at 4°C after 45 days.

## 6 Perspective for future application in olive tissue culture

*In vitro* tissue culture systems have demonstrated significant potential as tools for understanding and addressing challenges in plant growth and development, particularly under abiotic stress conditions. Research indicates that the effects of abiotic stress observed *in vitro* often closely mimic those experienced in natural environments, making these systems valuable for studying water and salt stress in plants (Pérez-Clemente and Gómez-Cadenas, 2012; Silvestri et al., 2017; Bashir et al., 2021). For olive plants, studies by Bashir et al. (2021) and Silvestri et al. (2017) investigated the impact of NaCl-induced salt stress and PEG-induced drought stress on a salt-tolerant cultivar and two transgenic lines overexpressing the tobacco osmotin gene. Beyond their role in stress studies, micropropagation and tissue culture provide versatile tools for biotechnological and genetic advancements in olive trees. For over three decades, these technologies have been utilized for various purposes, including thermotherapy for virus eradication, embryo rescue to accelerate breeding and enable interspecific crosses, and the induction of haploidy and polyploidy. Furthermore, they support somaclonal variation, somatic hybridization, and genetic engineering (Rugini et al., 2016). These methods are gaining renewed relevance with the advent of Next-Generation Breeding Strategies, such as genome editing. In olive trees, a persistent challenge lies in efficient regeneration through adventitious shoot organogenesis, somatic embryogenesis, and protoplast technology. Although almost underexplored in olives, these *in vitro*-mediated approaches are garnering increased interest due to their potential, aligning with evolving breeding and biotechnological goals.
